# The use of topical antiseptic solutions in the healing process of post-surgical skin wounds in dogs (*Canis lupus familiaris*)

**DOI:** 10.1590/acb400525

**Published:** 2024-01-13

**Authors:** Thomás Souza e Silva, Soke Gninlome Cedril Hounkonnou, Hiasmin Marcia de Souza Lima, Joana Luiza Crispiano Cunha Santos, Fábio de Souza Mendonça, Rinaldo Florencio-Silva, Ricardo dos Santos Simões, Fabrício Bezerra da Sá, Joaquim Evêncio-Neto

**Affiliations:** 1 Universidade Federal Rural de Pernambuco – Programa de Pós-Graduação em Medicina Veterinária – Departamento de Morfologia e Fisiologia Animal – Recife (PE) – Brazil.; 2 Universidade Federal Rural de Pernambuco – Departamento de Morfologia e Fisiologia Animal – Laboratório de Histologia – Recife (PE) – Brazil.; 3 Universidade Federal Rural de Pernambuco – Departamento de Morfologia e Fisiologia Animal – Recife (PE) – Brazil.; 4 Universidade Federal Rural de Pernambuco – Departamento de Morfologia e Fisiologia Animal – Recife (PE) – Brazil.; 5 Universidade Federal de São Paulo – Escola Paulista de Medicina – Departamento de Morfologia e Genética – Disciplina de Histologia e Biologia Estrutural - São Paulo (SP) – Brazil.; 6 Universidade de São Paulo – Faculdade de Medicina – Hospital das Clínicas – São Paulo (SP) – Brazil.

**Keywords:** General Surgery, Infections, Skin, Drug Resistance

## Abstract

**Purpose:**

To evaluate whether the effectiveness of topical antiseptic solutions in restoring skin continuity solutions is related to their antimicrobial action or to their action in maintaining moisture, in dogs undergoing elective surgeries.

**Methods:**

Forty dogs, 20 males and 20 females, underwent orchiectomy and oophorectomy, respectively. Thereafter, the animals were allocated into four groups (n = 5) and treated with different topical solutions: polyhexanide 0.1% (G1), chlorhexidine digluconate 0.05% (G2), povidone-iodine 0.1% (G3), and sodium chloride 0.9% (control). Macroscopically evaluation of wounds on days 1, 5, and 10 after procedures, and hematological parameters were performed. Data were subjected to statistical analysis (*p* 0.05)

**Results:**

No significant differences in the macroscopic evaluation of the wounds and hematological parameters were observed among groups, in the healing of clean elective surgical wounds. However, applying a topical solution dressing to maintain a moist wound promoted adequate tissue healing.

**Conclusion:**

In our experimental conditions, moisture provided by topical antiseptic solutions is an important factor compared to the antiseptic activity of these solutions, for skin wound healing in post-surgical dressings resulting from clean, short-term elective surgeries in male and female dogs. Moreover, the type of topical antiseptic solution used does not influence the development of post-surgical infection.

## Introduction

The skin is the largest organ in the animal body and is one of the main structures of the integumentary system. It is composed of the epidermis, dermis, and appendages (hair follicles, sebaceous glands, sweat glands, and nails). The skin performs important functions such as protecting the body against mechanical, physical, chemical, and harmful biological agents present in the environment, in addition to acting along with the peripheral nervous system to perceive pressure, pain, heat, and cold. Furthermore, the skin regulates the storage and excretion of water, electrolytes, vitamins, lipids, and thermoregulation^1^^,^ 2.

Skin continuity solutions have several etiologies, with surgeries and high-energy traumas, such as being run over, representing the main causes^3^^,^ 4. Therefore, when the skin is devitalized, the wound healing phenomenon is established. The restructuring of the integumentary system is a combination of physical, chemical, and cellular processes with the purpose of repairing the tissue in order to reestablish skin functions^5^.

Better healing occurs when the wound microenvironment is moist, as this promotes improved and selective autolytic debridement. In order for the wound to remain moist, solutions must remain in contact with it through the use of protections such as dressings. In environment exposed wounds, the healing process may be delayed due to greater formation of scabs, hindering cell migration through the devitalized tissue. Scabs rarely form in moist wounds or appear in smaller volumes, facilitating the penetration of topical medications, in addition to improving wound repair^6^.

Injuries to the integumentary system caused by surgical procedures require special attention, as they can develop complications, especially infections, which delay tissue repair and can progress to systemic conditions. Therefore, there are topical antiseptic solutions that can be used during surgical wound healing to optimize its restoration, in addition to combating infectious agents, resulting in the early return of its function^5^. Furthermore, topical antiseptic solutions have the advantage of providing two properties that help improve skin repair. One advantage is to keep the skin moist, the other one is to prevent the proliferation of pathogenic microorganisms in skin continuity solutions6. However, it is still unclear whether those beneficial effects are due to their antimicrobial action or to their action in keeping the skin moist during surgical wound healing. 

Thus, this study was designed to evaluate whether the effectiveness of topical antiseptic solutions in restoring skin continuity solutions after elective surgeries is related to their antimicrobial action or to their action in maintaining moisture during skin repair. It was carried out in the absence of antibiotic therapy as a way of preventing post-surgical infection in canine animals (*Canis lupus familiaris*) undergoing elective orchiectomy and ovariohysterectomy surgeries.

## Methods

### Ethics Committee

The project was approved by the Ethics Committee on the Use of Animals (CEUA) of the Universidade Federal Rural de Pernambuco (UFRPE), and registered under no. 949629062. All experimental procedures followed the principles of animal care and welfare determined by the CEUA of UFRPE, and good practice of laboratory protocols and quality assurance methods were also carried out.

### Commercial samples of topical solutions

Four topical solutions approved in Brazil for use on wounds and antisepsis procedures were used in the present study and are described in Table 1. The solutions were adapted for use in the research were packaged in spray bottles chemically sterilized with benzalkonium chloride (Herbalvet T.A.), at the dilution of 1:500. The spray bottles had the total volume of 100 mL for later distribution to the dog caregivers. The chlorhexidine digluconate solution had an initial concentration of 2%, so it was diluted with sterile 0.9% sodium chloride (NaCl) at a ratio of 1:40 to reach a concentration of 0.05%. G3 had 10% polyvidone iodine as a stock solution; therefore, it was diluted at a ratio of 1:100 to obtain a concentration of 0.1%.

**Table 1:** Topical solutions used in the present study.

Group	Product	Active ingredient	Properties
G1	Pielsana	Polyhexamethylene biguanide hydrochloride (0.1%)	Broad spectrum of action in controlling bacteria, fungi and viruses
G2	Riohex	Chlorhexidine digluconate solution with surfactants (2%)	It remains active in the presence of organic matter and has effective action against gram-positive and negative bacteria
G3	Riodeine	Povidone-iodine (10%)	Active against all forms of non-spore-forming bacteria, fungi and viruses
C	Saline solution	Sodium chloride (0.9%)	No proven antimicrobial action

Source: Elaborated by the authors.

### Post-surgical wounds in dogs

Male dogs underwent orchiectomies using the pre-scrotal technique, whereby each testicle was pulled to the pre-scrotal region and the incision made above the testicle in the median raphe. Females underwent ovariohysterectomies (OH) through the ventral midline, with the incision made caudal to the umbilical scar. After surgical procedures, the solutions were placed in contact with the wounds by soaking the liquid in sterile gauze in two layers, one layer moist with the solution, and the other one dry on top, in order to allow fixation to the skin using micropore tape. The dressings were applied by the dog caregivers at home. In order to perform vascular ligations, sutures to close the abdominal cavity and eliminate dead space, 2-0 mononylon sutures were used. Regarding abdominoplasty (musculature) in females, the Sultan suture pattern was used. To close the skin in both males and females, the Donatti suture pattern was used with anchoring in the Alba line or subcutaneously, in order to reduce dead space.

### Macroscopic evaluations of wounds

The wounds were evaluated by the same evaluators (two independent evaluators blinded to the study) throughout the experiment, with emphasis on macroscopic aspects, as follows: presence of hyperemia, secretion, crusts, and healing. It is worth noticing that the wounds were quantified by scores according to their appearance during inspection, as described ahead (Tables 2–5).

**Table 2:** Assessment of the macroscopic degree of hyperemia in post-surgical wounds.

Score	Parameter
0	Absent (wounds with skin color)
1	Low hyperemia (pink wounds)
2	Median hyperemia (pinkish-reddish wounds)
3	Intense hyperemia (deep red wounds)

Source: Elaborated by the authors.

**Table 3:** Assessment of the macroscopic degree of secretion in post-surgical wounds.

Score	Parameter
0	Absent (no secretion from the wound)
1	Mild (presence of secretion in some areas of the incision)
2	Moderate (presence of secretion throughout the incision)
3	Intense (presence of secretion throughout the incision and at the time of evaluation).

Source: Elaborated by the authors.

**Table 4:** Assessment of the macroscopic degree of the presence of crusts in post-surgical wounds.

Score	Parameter
0	Absent (no crust at the incision line)
1	Low (presence of some crusts at the incision line)
2	Medium (presence of crusts along the entire incision line)
3	High (presence of crusts on the incision line and surrounding areas)

Source: Elaborated by the authors.

**Table 5:** Assessment of the macroscopic degree of healing in post-surgical wounds.

Score	Parameter
0	Absent (incision in initial post-surgical appearance)
1	Incomplete (presence of one of the parameters previously evaluated, hyperemia, secretion or crusts)
2	Complete (incision with absence of hyperemia, crusts and secretion, presenting appearance of the skin without alterations)

Source: Elaborated by the authors.

### Assessment of systemic infection

The presence of systemic infection was analyzed by physical evaluation, in addition to complementation through blood counts five days after surgery. The physical evaluation consisted of checking the following parameters: temperature by rectal measurement; capillary perfusion time in the oral mucosa; hydration verification by skin turgor; heart rate; respiratory rate; in addition to the color of the ocular and oral mucosa. The presence of infection was quantified by scores according to the results of hematological exams and clinical evaluation of the animals as described ahead (Table 6).

**Table 6:** Assessment of post-surgical infection rate.

Score	Parameter
0	Absent (absence of hyperemia, crusts and secretion in a score greater than or equal to 2, and no hematological or clinical changes)
1	Present (presence of secretion, crusts, hyperemia with a score greater than or equal to 2, in addition to the presence of hematological and clinical changes)

Source: Elaborated by the authors.

### Statistical analysis

The scores (0, 1, 2 or 3) attributed for the evaluation of the macroscopic degree of hyperemia, secretion, crusts, healing, in addition to the presence of infection on days 1, 5 and 10 were analyzed by the nonparametric Wilcoxon’s test, using the pairwise.wilcox.test() function. Significant differences between groups of topical solutions, sex of animals and days of development of clinical parameters were set at *p* < 0.05. Regarding blood counts, statistics were performed via analysis of variance (ANOVA) using mixed linear models in a 4×2 experimental design (four topical solutions and two sexes) in repeated measures over time (blood count before and after surgery), with the factor’s topical solutions, sex and time composing the fixed factors, and the animals composing the random factor. The means were compared by 95% confidence interval using the Tukey’s test (*p* < 0.05). All statistical analyses were performed using the R Software^7^.

## Results

Elective OH surgeries and orchiectomies were performed on 40 canine animals, 20 males and 20 females, during the period from August 2021 to October 2022. The average age of the animals was 2.7 years old (standard deviation of 1.72 years), with an average weight of 13.61 kg (standard deviation of 8.99 kg). A breed standard was not established for the experiment, with most animals (55%) as mixed-breed dogs (22/40). However, animals of other breeds were also part of the sample such as Shih-Tzu 15% (6/40), American Pit Bull Terrier 7.5% (3/40), Pinscher 5% (2/40), Yorkshire Terrier 2.5% (1/40), German Shepherd 2.5% (1/40), Labrador Retriever 2.5% (1/40), German Spitz-Toy 2.5% (1/40), Chow-Chow 2.5% (1/40), Maltese 2.5% (1/40), and French Bulldog 2.5% (1/40).

In the initial analysis of the results, the scores obtained after the assessments were subjected to non-parametric statistical evaluation, using the median values. To calculate the medians, the variables of sex and days were included, enabling a general comparison between the groups (Table 7).

**Table 7:** Data from medians of macroscopic evaluations of wounds in canine animals, after using different topical solutions for the treatment of post-surgical wounds resulting from elective castrations.

Groups	Hyperemia	Secretion	Crusts	Healing	Infection
C	0	0	0	1	0
G1	0	0	0	1	0
G2	1	0	0	1	0
G3	0	0	0	1	0

C, Control; G1, Polyhexanide 0.1%; G2, Chlorhexidine Digluconate 0.05% and G3, Povidone-iodine 0.1%. For hyperemia, the value 0 means absent (wounds with skin coloration), and the value 1 means low (pink wounds). Regarding secretion, the value 0 means absent (no secretion in the wound). Regarding the parameter of the presence of crusts, the value 0 means absent (no crust in the incision line). Regarding healing, the value 1 means incomplete (presence of one of the parameters observed previously, hyperemia, secretion or crusts above 1). For infection, the value 0 means absent (absence of hyperemia, crusts and secretion in a score greater than or equal to 2, and no hematological and clinical alterations). Source: Elaborated by the authors.

**Figure 1:**
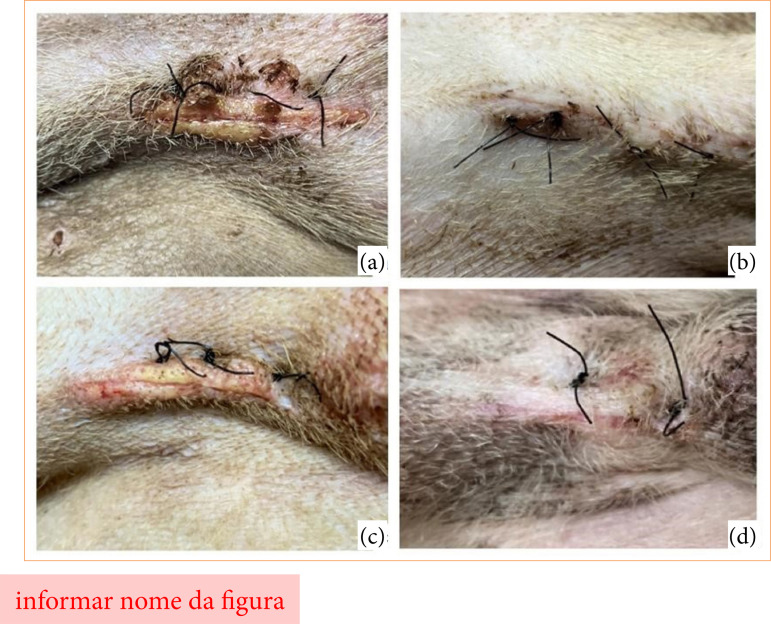
**(a)** Dog from G1 showing signs of licking and biting at the wound site five days after surgery. Note the presence of slightly hyperemic edges and the yellowish coloration of part of the incised edges. Crusts formed around the incision due to increased secretion are also noticed. **(b)** Dog from G1 with the same evaluation time as the previous animal, but with adequate management of the post-surgical wound, that is, no access to the wound by the patient and provision of moisture with the topical solution. **(c)** Dog from G2 showing signs of licking and biting at the wound site five days after surgery. The presence of slightly hyperemic edges is noticeable, as well as the yellowish coloration of part of the incised edges similar to those of the patient from G1. **(d)** Dog from G2, with the same evaluation time as the previous animal, but with adequate management of the post-surgical wound.

There were no significant differences between the groups with the different topical solutions in relation to the macroscopic aspects of the wounds, in addition to the clinical and hematological evaluation to confirm infection. However, G2 presented a greater degree of hyperemia in the region of the incision line as compared to the other groups. When analyzing the variables in Table 7, two more tables were created, one comparing the scores between sexes and the other between days. Regarding sex, there were no significant differences between males and females for all the parameters evaluated (presence of hyperemia, secretion, crusts, healing and infection) (Fig. 1; Tables 8 and 9).

**Table 8:** Data from medians of macroscopic evaluations of wounds in canine animals, after using different topical solutions for the treatment of post-surgical wounds resulting from elective castrations.

Groups	Hyperemia	Secretion	Crusts	Healing	Infection
C	0	0	0	1	0
G1	0	0	0	1	0
G2	1	0	0	1	0
G3	0	0	0	1	0

C: control; G1: polyhexanide 0.1%; G2: chlorhexidine digluconate 0.05%; G3: povidone-iodine 0.1%. For hyperemia, the value 0 means absent (wounds with skin coloration), and the value 1 means low (pink wounds). Regarding secretion, the value 0 means absent (no secretion in the wound). Regarding the parameter of the presence of crusts, the value 0 means absent (no crust in the incision line). Regarding healing, the value 1 means incomplete (presence of one of the parameters observed previously, hyperemia, secretion or crusts above 1). For infection, the value 0 means absent (absence of hyperemia, crusts and secretion in a score greater than or equal to 2, and no hematological and clinical alterations). Source: Elaborated by the authors.

**Table 9:** Data from macroscopic evaluations of wounds in canine animals, comparing the values obtained after evaluations of males and females.

Sex	Hyperemia	Secretion	Crusts	Healing	Infection
Female	0	0	0	1	0
Male	0	0	0	1	0

Regarding hyperemia, the value 0 means absent (wounds with skin color). For secretion, the value 0 means absent (no secretion in the wound). Regarding the parameter of the presence of crusts, the value 0 means absent (no crust in the incision line). For healing, the value 1 means incomplete (presence of one of the parameters observed previously, hyperemia, secretion or crusts above 1). Regarding infection, the value 0 means absent (absence of hyperemia, crusts and secretion in a score greater than or equal to 2, and no hematological and clinical alterations). Source: Elaborated by the authors.

Regarding days 1, 5, and 10 after surgery, there were no significant differences in relation to the presence of hyperemia, secretion, and infection. Nonetheless, regarding the presence of crusts, five days after surgery the animals presented more crusts in the incision line compared to days 1 and 10. Furthermore, the healing rate was higher on day 10 compared to days 1 and 5 (Table 10).

**Table 10:** Data from the medians of the macroscopic evaluations of the wounds of canine animals, comparing the values obtained between the evaluation days (1, 5 and 10) after the surgeries.

Days	Hyperemia	Secretion	Crusts	Healing	Infection
1	0	0	0	1	0
5	0	0	1	1	0
10	0	0	0	2	0

For hyperemia, the value 0 means absent (wounds with skin color). Regarding secretion, the value 0 means absent (no secretion in the wound). Regarding the parameter of the presence of crusts, the value 0 means absent (no crust in the incision line) and the value 1 means low (presence of some crusts in the incision line). Regarding healing, the value 1 means incomplete (presence of one of the parameters observed previously, hyperemia, secretion or crusts above 1), and the value 2 means complete (incision with absence of hyperemia, crusts and secretion, presenting an appearance of the skin without alterations). For infection, the value 0 means absent (absence of hyperemia, crusts and secretion in a score greater than or equal to 2, and no hematological and clinical alterations). Source: Elaborated by the authors.

**Figure 2:**
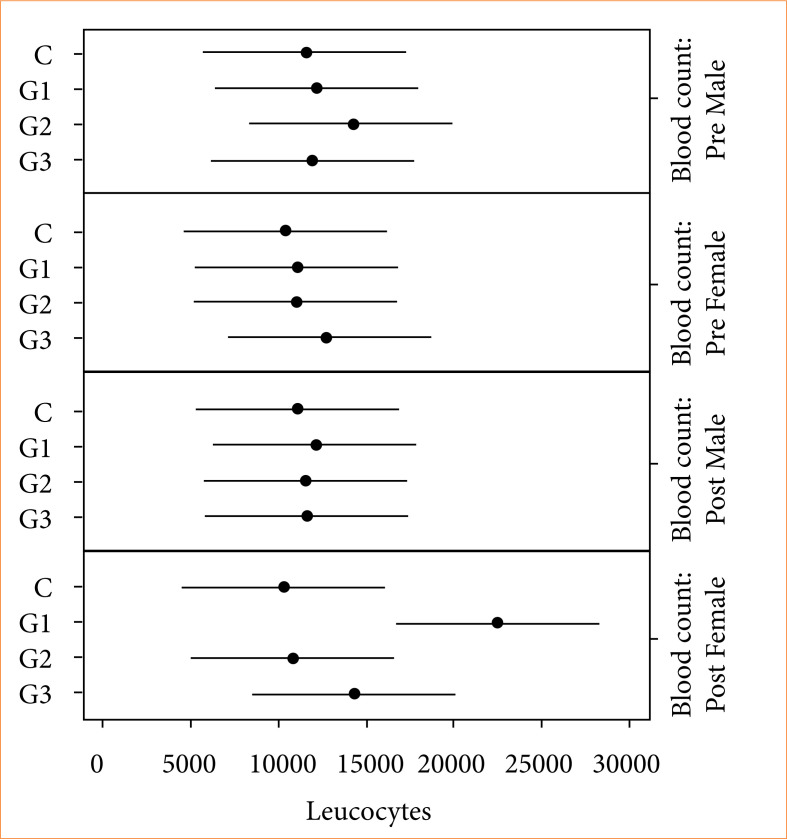
Distribution of the means of total leukocytes in animals of both sexes under different types of topical solutions. The parameters were obtained before surgery (pre-surgical blood count) and after surgeries using topical solutions (post-surgical blood count). Horizontal bars indicate the 95% confidence interval for the mean. Bars that do not overlap vertically indicate a significant difference between the means (Tukey; p < 0.05).

After macroscopic evaluations, the average values of the pre- and post-surgical hematological exams were calculated, with emphasis on the leukogram, in order to complement the physical evaluation for the diagnosis of post-surgical infection. Therefore, the average values of total leukocytes with standard deviation were calculated, in addition to segmented neutrophils, eosinophils, lymphocytes, and monocytes. The average of total leukocytes was similar among all topical solutions. However, there was a significant increase in the average of the post-surgical hemogram of females from G1. Furthermore, in the same group, the confidence interval of G1 was greater in relation to control and G2, but it was similar to G3 (Fig. 2).

C: control group; G1: polyhexanide 0.1%; G2: chlorhexidine digluconate 0.05%; G3: povidone-iodine 0.1%. Source: Elaborated by the authors.

## Discussion

The animals of the G1 group did not show significant differences in the macroscopic parameters of the wounds or presence of infection, in relation to the other groups. Meanwhile, other authors have demonstrated that gauzes moistened with polyhexanide inhibits bacterial growth in culture media, mainly of gram-positive bacteria^8^. However, in the present study, the antiseptic action of this solution was not necessary for wounds resulting from elective and clean surgeries, since the solution of the control group demonstrated the same efficacy.

Meanwhile, significant differences were not observed in the macroscopic parameters of the wounds or presence of infection in the G2 group, as compared to the other groups. Similar to what was previously described for polyhexanide, it has been shown that chlorhexidine can be used effectively for infected wounds, and, thus, clean wounds may not obtain the full benefits of this solution^9^. However, a significant effect of 0.05% chlorhexidine on healing by secondary intention of clean skin wounds in dogs was reported. Therefore, it has been assumed that chlorhexidine has an important role in antibacterial activity when compared to the control group, although the healing speed between the control and chlorhexidine groups were similar^9^^,^ 10. Meanwhile, the wounds in the current study were healed by primary intention. This may have favored the non-contamination and uselessness of the antibacterial effect of the chlorhexidine solution, leading to a result similar to the results of the control group.

On the other hand, no significant differences were observed in the macroscopic parameters of the wounds or presence of infection in group G3, compared with the remaining groups. Researchers compared the antibacterial activity of 0.05% chlorhexidine with 0.1% povidone-iodine and saline as a control has been compared, in wounds healed by secondary intention performed surgically. It was concluded that chlorhexidine presented a better antibacterial effect and with prolonged action, for up to 6 hours, albeit the wound healing area was similar between the solutions. Consequently, the antimicrobial effect of topical solutions may be dispensable in clean surgical wounds healed by primary intention^11^.

OH surgery is more invasive than orchiectomy and requires greater precision in surgical technique. Therefore, it presents a greater chance of infections in the postoperative period^12^^,^ 13. Despite the differences in surgical technique and risks inherent to the procedures, no significant difference was observed in the healing time and macroscopic parameters of post-surgical wounds in male and female dogs. Therefore, the health status of the patients, adequate surgical technique, ideal wound environment, and postoperative care act as key factors for better wound healing^14^.

Moisture at the wound site is an essential factor in post-surgical management^6^. Therefore, in patients who presented better healing, the owners reported that they were able to apply the dressing as recommended. Thus, these wounds did not present crusts at the incision line, in addition to a macroscopic appearance similar to the adjacent skin that was not injured; moreover, it was possible to observe healing in the maturation phase from the fifth day of evaluation. In contrast, it has been reported that the healing time of surgical skin wounds in canines lasts approximately ten days, if there are no complications^15^.

**Figure 3:**
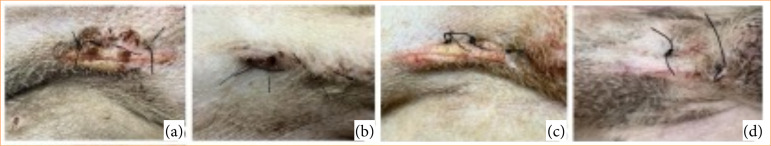
**(a)** Dog from G1 showing signs of licking and biting at the wound site five days after surgery. It is possible to observe the presence of slightly hyperemic edges, in addition to the yellowish coloration of part of the incised edges. It is worth noticing that crusts formed around the incision due to increased secretion. **(b)** Dog from G1, with the same evaluation time as the previous animal, but with adequate management of the post-surgical wound, that is, no access to the wound by the patient and provision of moisture with the topical solution. **(c)** Dog from G2 showing signs of licking and biting at the wound site five days after surgery. The presence of slightly hyperemic edges is noticeable, in addition to the yellowish coloration of part of the incised edges, characteristics similar to those of the patient from G1. **(d)** Dog from G2, with the same evaluation time as the previous animal, but with adequate management of the post-surgical wound.

Source: Elaborated by the authors.

The presence of crusts is common in dry wounds without any topical solution^6^. Consequently, in our study it was possible to identify that the caregivers were not following the post-surgical recommendations, due to the macroscopic appearance of the wounds (presence of crusts) during the evaluation periods. In addition, the presence of crusts increases the time for completion of wound healing, since they hinder cell migration through the incised dermis. However, the absence of scabs at the incision line may not always indicate a healthy environment for wound healing, since their absence, together with changes in the macroscopic parameters of the wound, may indicate signs of licking and/or biting of the wound. In this study, some of the dogs (15%, 6/40) presented, from the fifth day of evaluation onwards, absence of scabs, slight hyperemia at the edges of the incised skin, secretion in the wound, in addition to yellowish color at the edges (Fig. 3). These signs were standardized for animals that licked and/or bitten post-surgical wounds, requiring three or more of the signs mentioned above for confirmation, in addition to a history of difficulty in maintaining surgical clothing and an Elizabethan collar 24 hours a day.

Among the animals that participated in the study, 5% (2/40) showed signs of post-surgical infection, which belonged to groups G1 and G2 of females. The presence of post-surgical infection in elective clean surgeries has been described in 5.5% (85/1550) of dogs. Furthermore, most infections were established before seven days after surgery, similar to the present study^16^. A rate of 2.5% (27/1100) of post-surgical infection was also observed in clean elective surgeries, a lower percentage compared to the present study. However, this rate may have been reduced due to the use of antibiotics in most of the animals^17^.

Some factors intrinsic to surgical procedures may have contributed to the progression of the infectious process in the postoperative period. Among them, the duration of surgery stands out, since each hour of surgery doubles the risk of infection, due to the side effects of anesthesia such as hypothermia and hypotension, reducing the patient’s immune response. Thus, females presented a greater risk of infection progression, since the surgery lasted longer than in males. Therefore, this factor may have contributed to the non-progression of possible infections in these animals^16^^,^ 17.

Surgeries lasting longer than 120 minutes have a higher chance of developing infections, regardless of the use of antibiotics^16^. Furthermore, a lower infection rate is observed in clean surgeries in which the surgical time is less than 90 minutes. Furthermore, experiences from surgeons can also contribute to reducing infection, showing that time alone would not be a determining factor, but with great importance^17^. However, in the present study, the average time to perform OH in females was 45 minutes and for orchiectomies 17 minutes. In this perspective, the longer time to perform surgeries may have contributed to the development of complications in females.

On the other hand, the use of antibiotics prophylactically in surgeries by itself is not the determining factor for the occurrence or not of infection at the surgical site, as there are other related causes^18^. In the present study, a recurring complaint from owners was the difficulty in maintaining the Elizabethan collar and surgical clothing, especially in males, leading to the removal of the dressing, licking of the wounds, delayed healing and increased risk of infection. Postoperative care and the dog’s behavior at home may be also important in the development of these postoperative complications^15^. However, even with licking, infection did not occur in all patients, probably due to the healthy state of the patients and efficient immune response^19^. The lack of infections acquired in the surgical center in the current study may be also linked to good surgical practices in general (environment, apparatus, team), in addition to the preparation of the surgical site^9^.

The method chosen for skin antisepsis at the surgical site may also have contributed significantly to preventing infection. The use of 2% chlorhexidine gluconate as a degerminant followed by 0.5% alcoholic chlorhexidine gluconate resulted in the elimination of all superficial microorganisms on the skin, with no growth of colony-forming units in culture media, even after surgery. Thus, the degerminant solution was used for initial cleaning, removing excess superficial dirt, for 3 minutes, followed by the alcoholic solution for 1,5 minute to maintain the residual effect^19^.

It is worth noticing that, in the current scenario, veterinary medicine has a wide variety of antibiotics available for use in the treatment of infections. However, these drugs are often used recklessly and excessively, favoring the development of antibiotic-resistant microorganisms. In specific cases such as infected wounds or contaminated surgeries, topical solutions can be used as an alternative to reduce the use of antimicrobial drugs, favoring the reduction of the emergence of multidrug-resistant strains. A limitation of our study was not having performed a microbial activity to analyze which solution is the most effective for each group studied, which could provide interesting data to the present study. Future studies are needed to evaluate this subject.

## Conclusion

Based on our results and the conditions of our study, we concluded that moisture provided by topical antiseptic solutions is an important factor, compared to the antiseptic activity of these solutions, for skin wound healing in post-surgical dressings resulting from clean, short-term elective surgeries in male and female dogs. The type of topical antiseptic solution used does not influence the development of post-surgical infection, since the risk of infection is minimal with the use of the appropriate surgical technique, reduced procedure time, and properly prepared instruments and environment.

## Data Availability

Data sharing is not applicable.

## References

[1] Sorg H, Sorg CGG (2023). Skin wound healing: of players, patterns, and processes. Eur Surg Res.

[2] Peña OA, Martin P (2024). Cellular and molecular mechanisms of skin wound healing. Nat Rev Mol Cell Biol.

[3] Balomenos DB, Gouletsou PG, Galatos AD (2023). Evaluation of incisional wound healing in dogs after closure with staples or tissue glue and comparison to intradermal suture pattern. Animals (Basel).

[4] Intarapanich NP, McCobb EC, Reisman RW, Rozanski EA, Intarapanich PP (2016). Characterization and comparison of injuries caused by accidental and non-accidental blunt force trauma in dogs and cats. J Forensic Sci.

[5] Medeiros AC, Filho AMD (2016). Cicatrização das feridas cirúrgicas. J Surg Clin Res.

[6] Ousey K, Cutting KF, Rogers AA, Rippon MG (2016). The importance of hydration in wound healing: reinvigorating the clinical perspective. J Wound Care.

[7] R Core Team (2021). R: a language and environment for statistical computing.

[8] Lee WR, Tobias KM, Bemis DA, Rohrbach BW (2004). In vitro efficacy of a polyhexamethylene biguanide-impregnated gauze dressing against bacteria found in veterinary patients. Vet Surg.

[9] Lozier S, Pope E, Berg J (1992). Effects of four preparations of 0.05% chlorhexidine diacetate on wound healing in dogs. Vet Surg.

[10] Sanchez IR, Swaim SF, Nusbaum KE, Hale AS, Henderson RA, McGuire JA (1988). Effects of chlorhexidine diacetate and povidone-iodine on wound healing in dogs. Vet Surg.

[11] Adin CA (2011). Complications of ovariohysterectomy and orchiectomy in companion animals. Vet Clin North Am Small Anim Pract.

[12] Souza FW, Brun MV, Oliveira MT, Feranti JPS, Corrêa RKR, Idalêncio R, Duda NCB, Quadros AM, Huppes RR (2014). Ovariohisterectomia por videocirurgia (via NOTES vaginal hibrida), celiotomia ou miniceliotomia em cadelas. Ciência Rural.

[13] Plater BL, Lipscomb VJ (2020). Treatment and outcomes of ureter injuries due to ovariohysterectomy complications in cats and dogs. J Small Anim Pract.

[14] Khoo TX, Yates G, Chambers B, Ng J (2022). Wound healing complications following folded flap palatoplasty in brachycephalic dogs. Aust Vet J.

[15] Stetter J, Boge GS, Grönlund U, Bergström A (2021). Risk factors for surgical site infection associated with clean surgical procedures in dogs. Res Vet Sci.

[16] Vasseur PB, Levy J, Dowd E, Eliot J (1988). Surgical wound infection rates in dogs and cats. Data from a teaching hospital. Vet Surg.

[17] Sørensen TM, Scahill K, Ruperez JE, Olejnik M, Swinbourne F, Verwilghen DR, Nolff MC, Baines S, Marques C, Vilen A, Duarte EL, Dias M, Dewulf S, Wichtowska A, Valencia AC, Pelligand L, Broens EM, Toutain PL, Alishani M, Brennan ML, Weese JS, Jessen LR, Allerton F (2024). Antimicrobial prophylaxis in companion animal surgery: A scoping review for European Network for Optimization of Antimicrobial Therapy (ENOVAT) guidelines. Vet J.

[18] Dacanay SJ, Barber RM, Diehl KA, Myrna KE (2022). Incidence and risk factors for surgical site infection following enucleation in dogs. Front Vet Sci.

[19] Trajano SC, Aragão BB, Penaforte MA, Melo KD, Ferreira MSS, Oliveira JMB, Mota RA, Aleixo GAS (2021). Bacterial isolation and evaluation of antisepsis protocols of the operative field of bitches submitted to ovariohysterectomy. Braz J Vet Med.

