# ERRATA

**DOI:** 10.1590/acb400525.errata

**Published:** 2025-02-28

In the manuscript **The use of topical antiseptic solutions in the healing process of post-surgical skin wounds in dogs (*Canis lupus familiaris*)**, which DOI is: https://doi.org/10.1590/acb400525, published in the journal **Acta Cirúrgica Brasileira**, 2025, v40, e400525, page 5, in the title of Figure 1:

Instead of

**Figure 1** – XXXXX

Should be

**Figure 1– (a)** Dog from G1 showing signs of licking and biting at the wound site five days after surgery. Note the presence of slightly hyperemic edges and the yellowish coloration of part of the incised edges. Crusts formed around the incision due to increased secretion are also noticed. **(b)** Dog from G1 with the same evaluation time as the previous animal, but with adequate management of the post-surgical wound, that is, no access to the wound by the patient and provision of moisture with the topical solution. **(c)** Dog from G2 showing signs of licking and biting at the wound site five days after surgery. The presence of slightly hyperemic edges is noticeable, as well as the yellowish coloration of part of the incised edges similar to those of the patient from G1. **(d)** Dog from G2, with the same evaluation time as the previous animal, but with adequate management of the post-surgical wound.

